# Exploration beyond osteoarthritis: the association and mechanism of its related comorbidities

**DOI:** 10.3389/fendo.2024.1352671

**Published:** 2024-04-19

**Authors:** Bo Li, Zhenguo Yang, Yang Li, Jiuchao Zhang, Chengen Li, Naishan Lv

**Affiliations:** The Second Affiliated Hospital of Shandong University of Traditional Chinese Medicine (Shandong Hospital of integrated traditional Chinese and Western medicine), Jinan, China

**Keywords:** osteoarthritis, osteoporosis, sarcopenia, cardiovascular diseases, diabetes mellitus, dementia, Parkinson’s disease, mental disease

## Abstract

Osteoarthritis is the most prevalent age-related degenerative joint disease and a leading cause of pain and disability in aged people. Its etiology is multifaceted, involving factors such as biomechanics, pro-inflammatory mediators, genetics, and metabolism. Beyond its evident impact on joint functionality and the erosion of patients’ quality of life, OA exhibits symbiotic relationships with various systemic diseases, giving rise to various complications. This review reveals OA’s extensive impact, encompassing osteoporosis, sarcopenia, cardiovascular diseases, diabetes mellitus, neurological disorders, mental health, and even cancer. Shared inflammatory processes, genetic factors, and lifestyle elements link OA to these systemic conditions. Consequently, recognizing these connections and addressing them offers opportunities to enhance patient care and reduce the burden of associated diseases, emphasizing the need for a holistic approach to managing OA and its complications.

## Introduction

**Figure 1:**
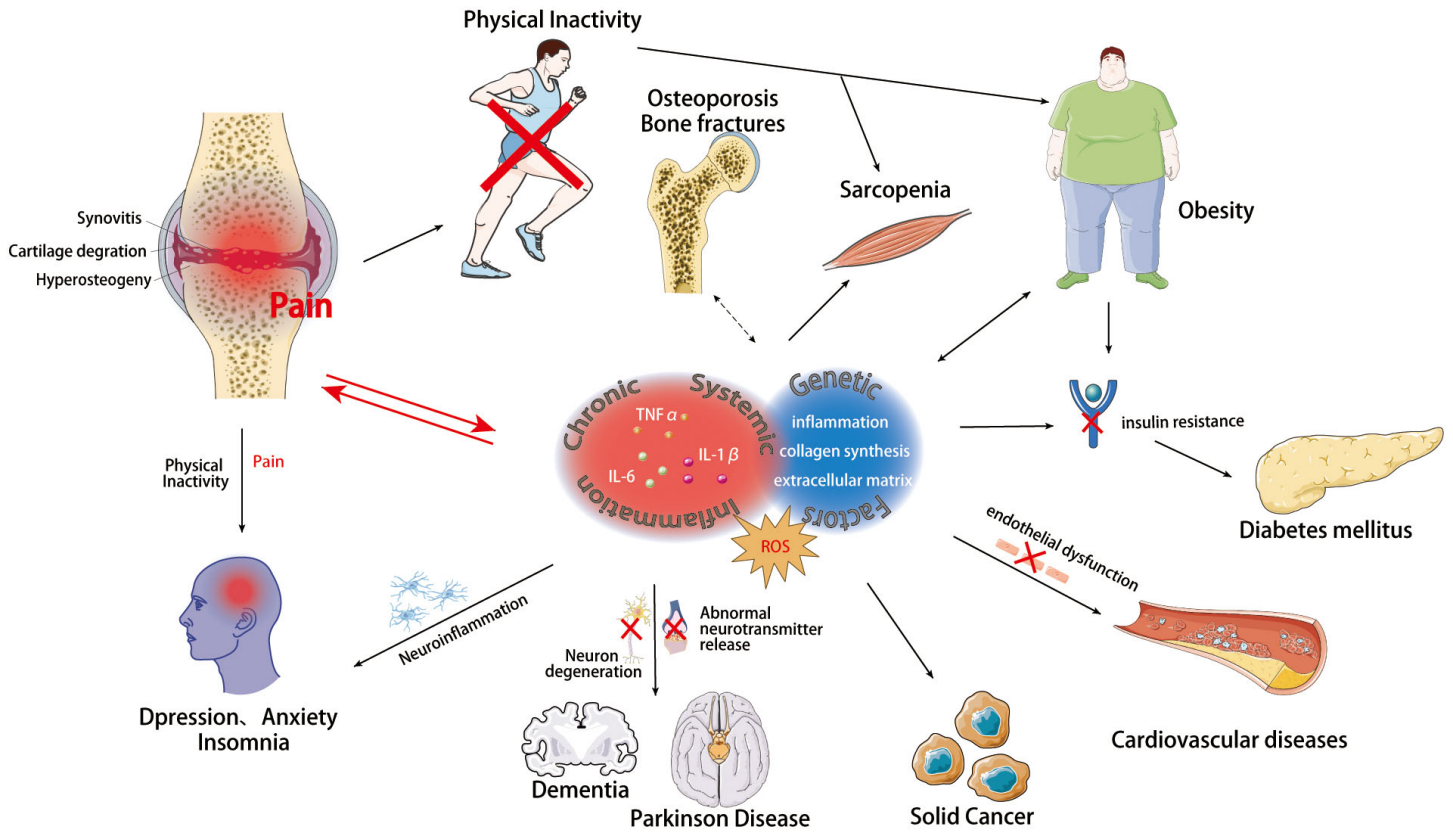
Chronic systemic inflammation, ROS, and genetic factors link osteoarthritis with multisystemic comorbidities mechanisms. Osteoarthritis, a chronic degenerative joint disease, involves structural changes in the hyaline articular cartilage, subchondral bone, ligaments, capsule, synovium, periarticular muscles, as well as meniscus degeneration and infrapatellar fat pad fibrosis and inflammation. There are intricate connections between OA and various systemic diseases, sharing common underlying mechanisms including Chronic Systemic Inflammation, ROS, and Genetic Influences. These mechanisms contribute to the pathogenesis of OA and serve as significant risk factors for multi-system comorbidities. Templates adapted to create this figure are freely available from Servier Medical Art (https://smart.servier.com/), licensed under CC BY 4.0 (https://creativecommons.org/licenses/by/4.0/).

**Figure 2:**
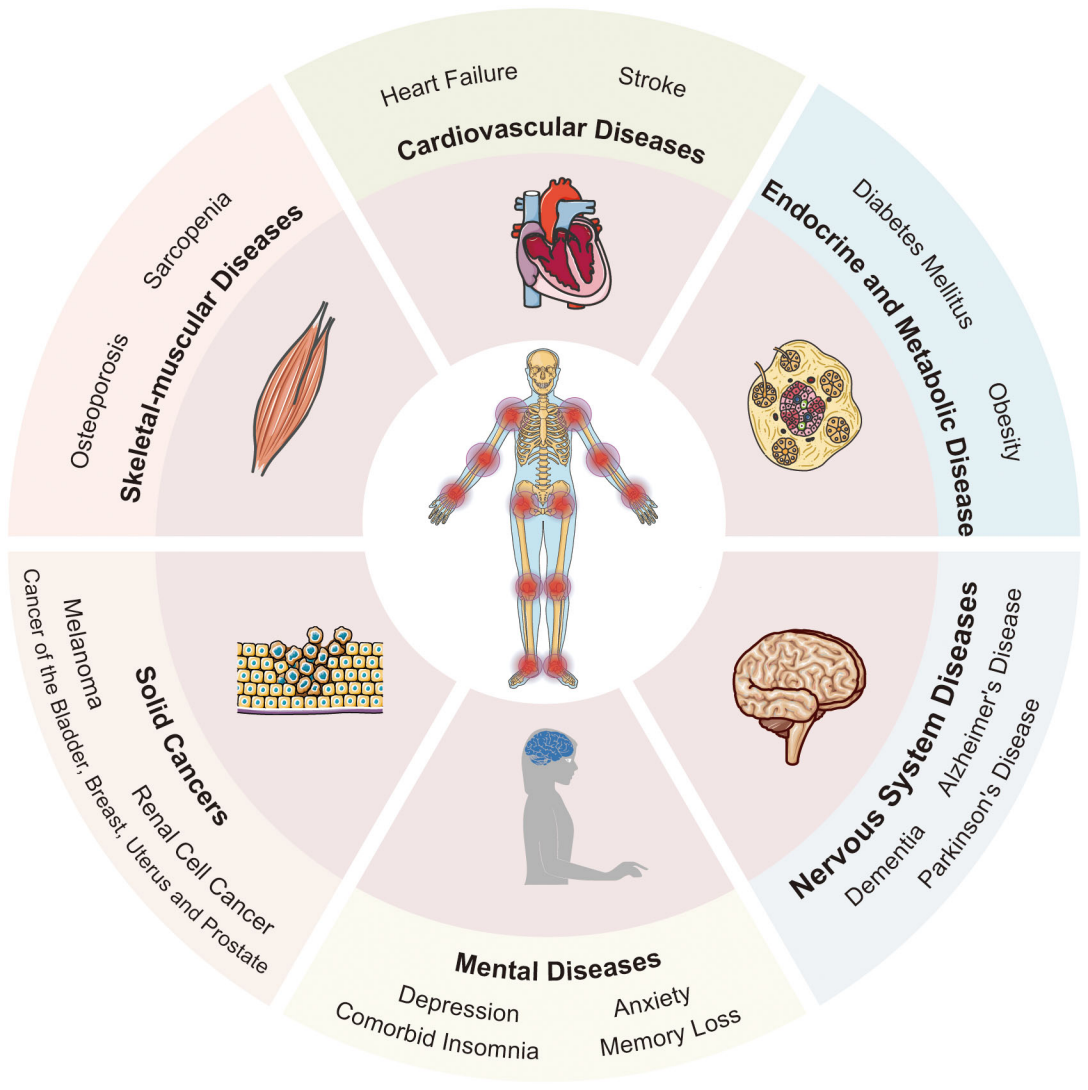
The multisystemic impact of osteoarthritis: a comprehensive overview. OA manifests symbiotic relationships with several systemic diseases, thereby giving rise to numerous comorbidities. These include skeletal-muscular diseases, cardiovascular diseases, endocrine and metabolic diseases, nervous system diseases, mental diseases, and solid cancers. Templates adapted to create this figure are freely available from Servier Medical Art (https://smart.servier.com/), licensed under CC BY 4.0 (https://creativecommons.org/licenses/by/4.0/).

Osteoarthritis (OA) is a chronic degenerative joint disease, impacting the entire joint (1). It arises from an imbalance between the repair and degradation of joint tissues. This leads to structural changes in the hyaline articular cartilage, subchondral bone, ligaments, capsule, synovium, and periarticular muscles (2, 3), such as degeneration and loss of articular cartilage, subchondral bone sclerosis, subchondral bone cyst, weakened and frayed tendons/ligaments/muscles, capsular fibrosis, synovial hyperplasia and inflammation of the synovitis membrane, as well as meniscus degeneration and infrapatellar fat pad fibrosis and inflammation (4, 5) (**Figure 1**). These alterations ultimately lead to joint failure (6). Structural damage in OA, referred to as structural OA, includes cartilage loss, osteophyte formation, subchondral bone changes, and meniscal alteration, etc, all of which can be visualized through MRI. Joint pain may accompany these alterations, indicating symptomatic OA (7). Being the most common joint disease and a leading cause of disability among older adults, OA affects approximately 7% of the global population, exceeding 500 million people worldwide, with women being disproportionately affected (8, 9). The prevalence of OA is expected to rise further, given the trends of an aging population and increasing rates of obesity, making it more widespread than in previous decades (10–13). Traditionally regarded as a localized joint disorder, emerging evidences have shed light on OA as a condition that exacerbates various comorbidities, underscoring its profound health implications (14). The association between OA and the subsequent development of diverse common conditions highlights an overarching burden that demands attention from the broader medical community. Therefore, there is an urgent need to thoroughly investigate the relationship between OA and its comorbidities. This review aims to offer a comprehensive perspective on OA management, emphasizing the holistic approach necessary to address both OA and its comorbidities effectively. By highlighting the importance of comorbidities in symptomatic OA individuals, we strive to contribute to the development of improved strategies for patient care, ultimately reducing the burden of related diseases. **Figure 2** visually represents the intricate interplay of OA with various systemic complications discussed in the subsequent sections of this paper.

## Methodology

For this review, an extensive search of the PubMed database and Google Scholar was conducted using the keywords “osteoarthritis” and “comorbidities”. Recent English-language original articles, meta-analyses, and systematic reviews focusing on prevalence or incidence and the risk ratio of OA comorbidity were included. Longitudinal studies were prioritized. Comorbidity was defined as any chronic condition coexisting with osteoarthritis, considering OA as the primary exposure, and outcomes were centered on the presence of comorbidities. We further screened for conditions closely linked to osteoarthritis and with a high prevalence, refining our search to systematically investigate the connections between OA and various health issues. This included exploring musculoskeletal, cardiovascular, endocrine-metabolic, and neurological diseases, such as osteoporosis, sarcopenia, cardiovascular diseases, obesity, diabetes mellitus, dementia, Parkinson’s disease, depression, insomnia, tumors, and more. Included studies were required to report relevant outcomes related to the prevalence, incidence, risk factors, pathophysiological mechanisms, clinical manifestations, or management strategies of comorbidities in individuals with osteoarthritis. Studies exclusively focusing on unrelated comorbidities, as well as those with insufficient data or inadequate sample sizes to draw meaningful conclusions, were excluded. Figures in this manuscript were made using Adobe Illustrator software.

## Emerging role of OA in other system disease

OA is a chronic degenerative joint disease that primarily affects the hands, knees, hips, and spine. However, its impact extends beyond the joints, profoundly affecting other organ systems and exacerbating the morbidity and mortality associated with these conditions (15). Recent studies have shown that the presence and frequency of comorbidities such as CVDs, obesity, type 2 diabetes, and depression were associated with lower levels of physical activity in patients with OA (16–18). Kamps et al.’s (19) study in the Netherlands utilized a primary care database to explore the risk of general practitioner - diagnosed comorbidities following hip or knee OA diagnosis. After analyzing 58 prevalent comorbidities while adjusting for age and sex, they found individuals with knee and hip OA exhibited an elevated risk of being diagnosed with 30 and 26 different comorbidities, respectively. These findings confirm the strong association between OA and the subsequent development of various common conditions. Furthermore, these comorbidities further exacerbate the decline in patients’ quality of life and increase the burden on healthcare systems (20, 21). Therefore, understanding the relationship and underlying mechanisms between OA and its associated complications is crucial for effectively managing the disease and improving patient outcomes.

## Pathogenesis of OA

The onset of OA can be triggered by an array of factors, encompassing genetic predisposition, mechanical stress, and inflammatory processes (22). While a single pathogenic explanation for all OA cases remains elusive, genetic factors, sex disparities, age-related changes, and immunological processes collectively contribute to its development (22). Genetic investigations have identified multiple risk alleles distributed across the genome, underscoring the polygenic nature of OA (23). Epigenetic modifications, such as DNA methylation and histone affecting gene expression and tissue integrity (24), involving various factors like inflammatory mediators (IL-1β, IL-8), and stress factors associated with reactive oxygen species (SOD2, iNOS) (25, 26). Additionally, genetic alterations in key pathways like the TGF-β superfamily (27), Wnt/β-catenin (28), Notch (29), and Indian Hedgehog (Ihh) (30) have been implicated in osteoarthritis (OA) progression by disturbing the delicate balance between anabolic and catabolic processes in articular cartilage. These responses entail the upregulation of inflammatory mediators, leading to the degradation of the cartilage extracellular matrix (ECM) through increased expression of matrix metalloproteinases (MMPs) (31) and a disintegrin and metalloproteinase with thrombospondin motifs (ADAMTSs) (32). Mechanical factors, such as trauma, joint instability, and abnormal joint alignment, can also render individuals susceptible to the condition (33). OA emerges as a consequence of intricate interplays between local biomechanical factors and systemic susceptibility (34). Cartilage degradation and loss advance when the biomechanical stresses surpass the inherent resilience of the osteochondral structure (34). From a biomechanical perspective, injuries to cartilage and bone weaken their ability to withstand abnormal loads, leading to more severe structural damage (34, 35). Fractures and joint misalignments, such as varus and valgus deformities, result in incongruent joint lines and mechanical axis deviation, altering joint load distribution (36–38). Meniscal injuries lead to abnormal load transmission, increasing peak loads on joint cartilage and accelerating the progression of knee osteoarthritis, with meniscal protrusions also increasing local loading (39–41). Ligament laxity and muscle weakness result in joint instability, increasing shear forces on joint surfaces, leading to cartilage damage and the progression of osteoarthritis (42–44). Inflammation assumes a pivotal role in OA, with an increasing recognition of synovitis as a key feature. Inflammatory cytokines, notably IL-1β, are prominently involved in OA pathogenesis, with increased release observed from synovial fibroblasts in affected patients (45). This increase contributes to the development of an inflammatory pattern, including the elevation of TNF-α and IL-6 (46, 47). In general, the inflammatory condition not only exerts local effects but also leads to a pro-inflammatory state that can easily evolve into systemic inflammation. Disease progression involves the degradation of articular cartilage and subchondral bone, with a shift towards catabolic processes that favor tissue breakdown over repair mechanisms (48). Furthermore, the innate and adaptive immune systems have been implicated in OA, with immune cells, including lymphocytes, potentially contributing to the disease’s development (49, 50), although the exact mechanisms remain to be fully elucidated (**Figure 1**).

## Risk factors of OA

OA (OA) risk factors can be broadly divided into person-level and joint-level factors (51). Person-level factors include: (i) Age: increasing age is a prominent risk factor for OA, attributed to cumulative exposures and age-related changes in joint structures (52). (ii) Gender: women not only have a higher likelihood of developing OA but also tend to experience more severe cases (53). (iii) Obesity and metabolic syndrome: obesity, especially around the knee joint, is a recognized risk factor. Additionally, recent literature suggests an association between OA and metabolic syndrome (54). (iv) Genetic: genetic factors also play a role, with over 80 genes implicated in OA’s development, including those related to vitamin D receptors and bone health (55). (v) Diet: several dietary factors, including low levels of vitamins D, C, and K, are suspected contributors to OA development (56, 57). Joint-level factors encompass: (i) Injury: knee injuries, particularly anterior cruciate ligament (ACL) rupture, can lead to early-onset knee OA (13). The prevalence increases when associated with damage to cartilage, subchondral bone, and other structures (58, 59). Furthermore, prolonged joint damage can result in decreased joint function and disability, significantly impacting patients’ quality of life and social functioning. (ii) Abnormal loading of the joints: repetitive joint use is associated with OA development (51). Knee OA is linked to occupations requiring squatting and kneeling, hip OA is linked to prolonged lifting and standing (60), and hand OA is more frequent in people with occupations requiring increased manual dexterity (51).

## OA and skeletal-muscular disease

Osteoporosis (OP) is a systemic disease characterized by low bone mass, microarchitectural deterioration and susceptible to fragility fractures (61). In a review of 40 studies, a prevalence of 21.7% of OP was reported among the elderly population worldwide (43), with expectations of further increases as the population continues to age. Both OA and OP were common and frequently-occurring diseases in the elderly, which seriously affect their quality of life (62). Does OA influence the occurrence and progression of OP or osteoporotic fractures? The verdict remains pending. A review of the literature suggests inversely relationship between OA and OP (63), or even perhaps a lack of direct correlation (64). However, when analyzed in individual bones, the bone mineral density (BMD) of the appendicular skeleton in OA-affected joints may decrease (65). An investigation conducted by M Güler-Yüksel revealed that progressive hand OA over a 2-year span is linked with accelerated metacarpal BMD loss (66). Furthermore, in a cohort encompassing 199 patients with hip and knee OA, the overall prevalence of OP at any site was found to be 23%, with an additional 43% of patients meeting osteopenic criteria according to World Health Organization standards (67). Interestingly, OP was more prevalent in the forearm (14%) compared to the lumbar spine (8.5%) and the proximal femur of the index side (8.2%) (67). Dongkeun Kim’s meta-analysis revealed intriguing insights, although the overall frequency of OP in OA participants remained similar, a notable exception surfaced in the lumbar spine, revealing a higher prevalence of OP in both men and women compared to matched controls (68). Therefore, it is conceivable that OA accompanies the onset of OP.

OA and OP are both bone disorders typically afflicting the elderly population, imposing significant burdens of morbidity and disability (69). Recent strides in the realm of osteoimmunology have shed light on a commonality: heightened bone loss occurs not only in OP but also in the early stages of OA (70). Moreover, inflammation has emerged as a shared risk factor for both OP and OA. Furthermore, an unfavorable body composition contributes to the genesis of these conditions (71, 72). However, the nature of this contribution differs: while a lower Body Mass Index escalates the risk of OP, obesity exacerbates OA development (73). This occurs through a dual mechanism: increased mechanical stress on weight-bearing joints and putative adverse effects stemming from specific adipose tissue-derived adipokines (74). Additionally, several factors influence the progression of both OA and OP, encompassing sex hormones, ethnicity, age, dietary components, genetic predispositions, and physical activity levels (75). Aging, in particular, serves as a predictive factor for radiographic OA, bone loss, OP, and susceptibility to fractures (76).

Sarcopenia, another age-related skeletal muscle disorder, entails the accelerated loss of muscle mass and function, which is associated with increased adverse outcomes, including falls, functional decline, frailty, and mortality (77). Previous research has suggested a complex, bidirectional relationship between muscle weakness and OA. According to Toda et al. (78), women in the early stages of knee OA often exhibit a decline in lower extremity lean body mass relative to their total body weight. Clinical evidence underscores the significance of quadriceps weakness in knee OA. Ikeda and colleagues (79) discovered that the quadriceps muscle cross-sectional area was, on average, 12% smaller in asymptomatic women with incident radiographic OA compared to age and body-mass-matched controls. Similarly, Quadriceps weakness has also been linked to the progression of knee OA, attributed to disuse atrophy due to the pain and disability common in knee OA (80).

A significant contributing factor is the pain and stiffness experienced in osteoarthritic joints, which often leads to reduced physical activity, even giving rise to sarcopenic obesity (81). Inflammatory cytokines play a pivotal role in OA. A direct correlation has been established between IL-6 plasma levels and sarcopenia (82). Inflammatory cytokines disrupt protein synthesis and degradation in muscles and articular cartilage, precipitating synovial inflammation, muscle loss, and cartilage damage. In a rat model of knee osteoarthritis (OA), heightened IL-1β expression was noted in the affected gastrocnemius muscle, coupled with a 10% reduction in cross-sectional area (83). Moreover, the inflammatory milieu of knee OA induces neural alterations, exacerbating muscle atrophy. Discovered by Cunha et al. (84)in a rat model, is the restructuring of the quadriceps neuromuscular junction associated with knee osteoarthritis, resulting in heightened expression of the atrophy protein MuRF-1 and accelerating muscle atrophy and strength decline. However, in other studies (85), no such association has been found. Perhaps, the multifactorial etiology of sarcopenia takes into account the possibility of an inflammatory role in its pathogenesis, while this connection has not yet been definitively demonstrated.

## OA and cardiovascular diseases

Cardiovascular diseases (CVDs), including coronary heart disease, hypertension, and stroke, stand as prominent contributors to global mortality, claiming over 17.6 million lives annually and projected to escalate to 23.6 million by 2030 (86). Notably, research has unveiled a significantly heightened prevalence of CVDs among individuals afflicted with OA, surpassing that observed in the general population. For instance, a comprehensive meta-analysis, encompassing data from 15 studies, further corroborated these findings, revealing a pooled prevalence of 38.4% for overall CVDs pathology in individuals with OA (87). A prospective longitudinal study shed light on the increased susceptibility of elderly men and adult women with OA to developing CVDs, particularly ischemic heart disease and congestive heart failure (88). Hypertension, a frequently encountered comorbidity in patients with OA, was found to increase the likelihood of developing hypertension by 13% in individuals with knee OA (89). Furthermore, a BMI-independent association between hypertension and radiographic knee OA was observed (90). Moreover, a Mendelian randomization study utilizing inverse variance weighted analysis demonstrated a significant impact of hip osteoarthritis(HOA) on the development of heart failure and stroke (91). Similarly, a recent study utilizing Mendelian Randomization in the UK Biobank unveiled a novel causal association between low systolic blood pressure (SBP) and the clinical diagnosis of OA (92).

Shared risk factors such as age, obesity, lack of physical activity, as well as inflammation and genetics, may contribute to the development of both OA and CVDs (93). Increasing evidence suggests that inflammation plays a role in the connection between OA and CVDs (94). Cytokines and inflammatory processes can contribute to the destruction of cartilage and endothelial cells, thereby promoting vascular inflammation and the development of atherosclerosis (95), which forms the basis of many CVDs. Furthermore, some evidence suggests that the association between OA and CVDs may be mediated by common genetic factors (96). Several genes have been identified to be associated with both conditions, including those involved in inflammation (97), lipid metabolism (98), and extracellular matrix (97). Additionally, it is worth noting that OA patients often require the use of nonsteroidal anti-inflammatory drugs(NSAIDs) and other medications, which may have adverse effects on the cardiovascular system (99). These findings emphasize the importance of comprehensive management of these conditions, including lifestyle modifications, pharmacological interventions, and regular monitoring of cardiovascular health in OA patients.

## OA and endocrine & metabolic diseases

Diabetes mellitus (DM) is a group of metabolic diseases characterized by hyperglycemia resulting from defects in insulin secretion, insulin action, or both (100). The global incidence of diabetes is predicted to reach 642 million adults by 2040 (101). Chronic hyperglycemia and insulin resistance have the potential to cause harm to chondrocytes and induce cellular apoptosis (102). Additionally, research indicates that OA may potentially induce the onset of DM. A more extensive population-based study reported a 30% prevalence of high blood glucose in OA patients, while non-OA individuals exhibited a 13% prevalence (103).These prior investigations predominantly focused on specific joint locations, such as the knee, hand, or unspecified OA-afflicted joints (104).Some longitudinal studies have assessed the risk of developing DM in OA patients. In a comprehensive 12-year follow-up study encompassing 19,089 OA patients and an equal-sized control cohort, OA emerged as a pivotal risk factor for DM onset, except for elderly males, even after meticulous adjustment for covariates such as obesity (105). Similarly, a large-scale, protracted tracking study averaging 5.16 years, substantiated the importance of knee and hip OA as significant predictive factors for DM events (106). Moreover, supplementary investigations propose a conceivable linkage between OA and DM, possibly associated with joint replacement surgery. In the most recent study, which employed baseline walking speed as a fundamental metric, the diabetes incidence rate was scrutinized among individuals with knee OA or those at risk of knee OA (107).This study underscored that during a 7-month follow-up period, the cumulative diabetes incidence rate surged to a remarkable 96%, accentuating that slowed walking speed potentially stands as a pivotal predictive indicator for diabetes events in knee OA patients and those at risk of knee OA.

Diabetes and OA exhibit a close molecular relationship. Elevated blood sugar levels, a hallmark of diabetes, play a pivotal role in triggering and activating various molecular pathways associated with the development of OA (108). This intricate process hinges on the expression of several byproducts of glucose metabolism, including advanced glycation end-products (AGEs), sorbitol, and diacylglycerol. The connection between diabetes and OA stems from the inflammatory cascade set in motion due to heightened production of pro-inflammatory cytokines. OA is often accompanied by a backdrop of chronic joint inflammation, initiating the release of various inflammatory mediators, including but not limited to tumor necrosis factor-alpha (TNF-α) and interleukin-1 (IL-1) (109). These cytokines have the potential to directly or indirectly contribute to the development of insulin resistance, a pivotal characteristic of type 2 diabetes. The burden of pain and compromised joint function in OA may significantly curtail an individual’s physical activity levels, potentially resulting in weight gain and subsequent obesity, a well-established risk factor for DM (110). Numerous studies have substantiated the role of obesity and DM in promoting the onset and progression of OA. Obesity subjects joints to increased mechanical loading, thereby augmenting the pressure on joints and leading to damage and degeneration of joint cartilage, ultimately culminating in OA (111).It is plausible that obesity plays a crucial intermediary role, linking diabetes and OA, given their shared risk factors.

OA is not only intricately linked with DM, but evidence suggests that disrupted lipid metabolism may contribute to the pathogenesis of OA. A meta-analysis of cohort and cross-sectional investigations has exposed a notable heightened risk of OA in the presence of dyslipidemia, demonstrating a stark contrast with its absence (112). Additionally, a meta-analysis has identified a markedly elevated prevalence of dyslipidemia among OA sufferers, reaching up to 30%, significantly surpassing the 8.0% incidence found in the general population without OA. Furthermore, it demonstrated that the likelihood of dyslipidemia is significantly heightened in OA patients, with a risk ratio of 1.98 (113). Epidemiological evidence indicates a correlation between hypercholesterolemia and OA, implicating cholesterol as a systemic risk factor (114). Experimental studies using ApoE-knockout mice and diet-induced hypercholesterolemic rats have demonstrated that elevated cholesterol levels can induce OA-like pathologies, characterized by cartilage degradation, osteophyte development, and alterations in subchondral bone structure (115). Furthermore, increased circulating levels of cholesterol and triglycerides, coupled with dysfunctional high-density lipoprotein (HDL), have been associated with exacerbated joint pathology and cartilage loss in knee OA, mediated by synovial inflammation, ectopic bone formation, and an increased prevalence of bone marrow lesions, which are significant sources of pain in OA patients (116, 117).

Additionally, emerging research suggests a potential link between OA and other endocrine disorders, including hypothyroidism and hyperthyroidism. Studies have highlighted a correlation between these thyroid conditions and the onset of knee pain, as well as the development of knee OA (118). Notably, patients with hypothyroidism exhibit thinner femoral cartilage at all measured sites compared to healthy individuals, and are at a higher risk for OA (119). Intriguingly, meta-analytical reviews have revealed a more extensive connection, indicating that the presence of autoimmune thyroid diseases (AITD) not only elevates the risk of clinically significant thyroid diseases but also correlates with a higher prevalence of OA, rheumatoid arthritis (RA), and other musculoskeletal conditions (119, 120). Thyroid hormones play a crucial role in the development, growth, and maintenance of bone throughout life, but new research also suggests a potential significance of thyroid hormones in the articular cartilage lining of joint surfaces (121–123). Therefore, the relationship and mechanisms between thyroid hormones and osteoarthritis warrant further investigation.

## OA and nervous system disease

The nervous system serves as the primary system for sensing and transmitting pain signals throughout the body. Recent research has indicated a higher prevalence of neuropathic pain (NP) in individuals with OA (124, 125), where OA may lead to stimulation or damage of nerve endings located within or surrounding the joints, resulting in persistent or intermittent pain (126). These pain-related manifestations could potentially prompt changes in neural excitability, alterations in neurotransmitter release, and reduced neural plasticity (127). These alterations, in turn, may pave the way for central sensitization, wherein pain is perceived or exacerbated even in the absence of external stimuli (128).

Furthermore, emerging evidence suggests that OA may exert an influence on degenerative diseases within the central nervous system (129). Dementia is a progressive neurodegenerative brain disorder, leading to cognitive impairment, disability, and death in the elderly population, standing out as one of the major causes of mortality in individuals afflicted with knee and hip OA (130). A recent nation-wide study conducted in Taiwan has unveiled a noteworthy association between OA and an increased risk of dementia (131). Individuals with OA showed a significantly elevated likelihood of being diagnosed with incident Alzheimer’s disease(AD) and related dementias following meticulous adjustments for various factors (18). Supporting these epidemiological findings, animal studies reported that OA may accelerate the progression and exacerbation of dementia by instigating peripheral inflammation and neuroinflammation (132). Parkinson’s disease (PD), the second most common neurodegenerative disorder, stems from dopaminergic neuron degeneration, leading to motor impairments and non-motor symptoms (133). Cohort studies in Danish (134) and Taiwanese (135) populations have linked OA with an increased risk of developing PD. A retrospective cohort study involving 260,224 UK patients revealed a significantly higher incidence of PD in those with OA, supported by adjusted Cox regression analyses showing a 1.82-fold increased risk (136).

The exact mechanisms responsible for the observed connection between OA and nervous system diseases remain partially elucidated. However, OA appears to contribute to cognitive decline and the development of dementia and PD through multiple pathways. The chronic pain associated with OA can significantly impact neurocognitive function (127), potentially leading to cognitive impairment. Moreover, both systemic and peripheral inflammation likely play a role in this relationship. Elevated levels of systemic markers such as Hs-CRP, IL-6, and other proinflammatory mediators have been implicated in the progression of both OA and cognitive impairment (137). Shared genes related to inflammation, cartilage homeostasis, and neuronal function further substantiate the intricate links between OA and neurodegenerative diseases (138). Concerning the connection between OA and PD, inflammation appears to be a significant risk factor contributing to the etiopathogenesis of both conditions (139, 140). Another shared risk factor for OA and PD is vitamin D deficiency, often a consequence of limited sun exposure and prevalent worldwide (141). Additionally, the association between OA and PD may involve various mediating factors, including physical inactivity (142) (such as failing to meet recommended physical activity levels) and depression (143).

## OA and mental disease

OA exerts a potentially profound impact on mental health, a relationship elucidated by insightful epidemiological investigations (144). OA patients exhibit a prominent elevation in depression and anxiety prevalence, with a comprehensive meta-analysis of 49 studies divulging a pooled prevalence of 19.9% for depressive symptoms and 21.3% for anxiety symptoms among those with OA (145). Longitudinal data reinforces this, demonstrating that individuals with multi-site, hip, or knee OA exhibit greater odds of developing depressive symptoms compared to those without OA (113). Concomitantly, OA is linked to heightened likelihood of suicidal ideation underscoring its mental health implications (146). Moreover, individuals with OA also commonly report comorbid insomnia (147), as the pain and discomfort linked to the condition impede restful sleep, engendering fatigue and diminished quality of life (148). Moreover, OA patients were nearly three times more prone to frequent memory loss attributed to sleep and mood disturbances (149).

This symbiotic link is underscored by bidirectional interactions, where OA-related chronic pain, functional limitations, and distress contribute to the development and exacerbation of mental health conditions (150), while psychological factors, in turn, can further exacerbate the pain and inflammation (151), perpetuating a vicious cycle and influencing the perception and management of OA. In a recent systematic review, depressive symptoms were highlighted as a potential barrier to physical activity for people with OA (149). Furthermore, shared pathways involving neuroinflammation, oxidative stress, and genetic predispositions seem to underpin this connection (152).

## OA and tumors

Tumors, embodying uncontrolled cellular proliferation and invasive growth have conventionally stood apart from OA due to their divergent clinical manifestations and traditional spheres of study. Insights have been provided from extensive population-based investigations, illuminating the intriguing interplay between knee or hip osteoarthritis (KHOA) and cancer-related outcomes. Notably, two comprehensive studies, conducted in the United States and Sweden, revealed diminished cancer-related mortality among individuals afflicted with KHOA (153, 154). In contrast, a distinct meta-analysis presented a contrasting perspective, indicating an elevated risk of solid cancers ranging from melanoma to renal cell cancer, and cancers affecting the bladder, breast, uterus, and prostate in individuals with KHOA (155).A sole precedent study, delving into site-specific cancer incidence among OA patients, reported unexpectedly lower incidences of colorectal, stomach, and lung cancers, while noting an elevated incidence of prostate cancer (156). This variance may be plausibly attributed to prolonged use of NSAIDs (156), elucidating the multifaceted nuances of the intricate relationship between OA and cancer.

OA and tumorigenesis share complex molecular pathways. Chronic inflammation, a hallmark of OA, plays a crucial role in tumor progression, fostering an environment conducive to carcinogenesis (157). Pro-inflammatory cytokines and matrix metalloproteinases secreted by the synovium and the associated immune system can promote proliferation, viability, and migratory abilities of tumor cell (158). Genetic susceptibility has emerged as a key determinant in both OA and tumorigenesis (159). Shared genetic variants that influence inflammation, tissue remodeling, and cellular response have been implicated in both conditions. Oxidative stress, often observed in OA, similarly contributes to tumor development (160), prompting consideration of potential overlaps in therapeutic strategies targeting oxidative stress in both conditions.

## Conclusion and future perspectives

OA stands as a chronic degenerative joint ailment characterized by articular cartilage injury, cartilage loss, and hyperosteogeny, impacting over 500 million individuals worldwide (161). This comprehensive review has explored the multifaceted landscape of OA and its intricate connections to various systemic diseases including skeletal-muscular diseases, CVDs, endocrine and metabolic diseases, nervous system diseases, mental diseases and tumors (**Figure 1**).

The significance of this review lies in its revelation of the extensive interconnections between OA and systemic diseases, as well as the recognition that OA frequently coexists with other systemic conditions, such as diabetes and obesity, in a bidirectional relationship. These findings underscore the need for a holistic approach to managing OA and its associated complications. It’s important for patients with OA and diverse complications to formulate the corresponding treatment strategy. Education about OA and treatment plans is crucial for patients. Regular cardiovascular checks are important, especially for those with obesity and diabetes. Effective diabetes management, emphasizing lifestyle changes and medication adherence, is vital. Additionally, addressing mental health issues is essential due to their high prevalence in OA patients. For those with neuropathic pain, specialized neurological care can improve their quality of life. Regular screenings for potential comorbidities, such as cancer, may be considered for long-standing OA or high-risk individuals. In addition, understanding the shared risk factors and pathogenesis, such as inflammation and genetics, provides an opportunity for more targeted and comprehensive interventions that can improve the quality of life for OA patients and potentially reduce the burden of associated systemic diseases.

Future prospects in this field are promising but require further research to elucidate the intricate mechanisms underlying the relationships between OA and systemic diseases. This necessitates exploring the potential therapeutic avenues for addressing both OA and its associated comorbidities. Furthermore, comprehensive patient care should encompass early screening, timely interventions, and a focus on improving overall health to attenuate the impact of OA on systemic health.

In conclusion, OA transcends its status as a joint disorder, extending its influence into multiple organ systems. Acknowledging these connections and addressing them through research and clinical practice promises to enhance the quality of life for the millions of individuals burdened by OA, as well as mitigate the broader systemic health consequences of the disease.

## Author contributions

BL: Writing – original draft. ZY: Writing – review & editing. YL: Writing – review & editing. JZ: Writing – review & editing. CL: Writing – review & editing. NL: Writing – review & editing.
